# Cannot ventilate, Can we intubate?

**DOI:** 10.4103/0019-5049.71025

**Published:** 2010

**Authors:** Sarita Sutrayya Swami, Shubhada Sunil Aphale, Sunil Bapat

**Affiliations:** Department of Anaesthesiology, Bharti Vidyapeeth Medical College, Pune - 411 043, Maharashtra, India

Sir,

Cannot ventilate, can we intubate? This question arose in our mind when we saw a neonate in NICU with big oral mass, diagnosed as congenital epulis.

Congenital epulis is a well-known lesion arising from the alveolar ridge in newborns. Histologically it is a granular cell tumour,[1] usually not associated with other major congenital anomalies except polydactyly and neurofibromatosis.[2]

This tumour is also called as ‘Neumann’s tumour’ as was described by him first in 1871.[2] Prenatal diagnosis of epulis has been reported in the medical literature.[3]

**Figure 1:**
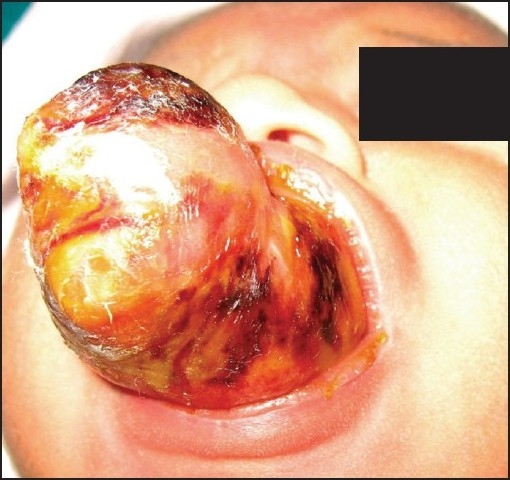
Mass occupying oral cavity

A 3 day old preterm female neonate weighing 1.9 kg, delivered vaginally at 36 weeks of pregnancy, presented to us with a large mass, occupying oral cavity [Figure 1] with difficulty in breast feeding; there was neither stridor nor respiratory embarrassment. This baby was scheduled for surgical excision of mass with primary diagnosis as congenital epulis.

**Figure 2:**
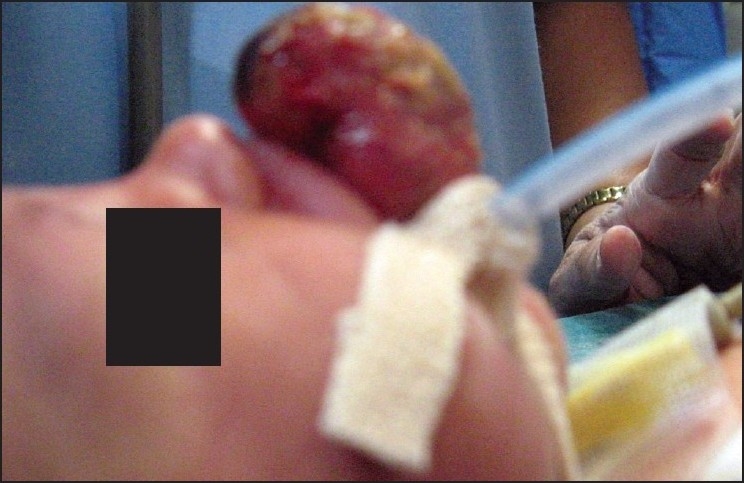
Neonate with ET tube *in situ*

After thorough preoperative examination and investigations which were within normal limits, baby was accepted as ASA grade II, an appropriate consent was taken. Due to big oral mass protruding outside the mouth, we could not ventilate with the mask. Therefore we did awake laryngoscopy with assistant displacing the mass; we could visualize the larynx easily. After pre-oxygenation with larger size mask, inhalational induction with sevoflurane was attempted. Once the baby became quiet, laryngoscopy was done carefully with an assistant gently displacing the mass. Laryngoscopy revealed a good view of vocal cords and trachea was intubated with un-cuffed ETT no. 3 successfully [Figure 2].

K. S. Canavan-Holliday and R. A. Lawson[2] also reported similar method of induction i.e. by spontaneous ventilation with halothane 0.5% and oxygen. They intubated baby on spontaneous ventilation with a Miller laryngoscope blade with an assistant gently pulling the epulis aside.

Merrett S.J., Crawford P.J.[4] reported that the excision of small masses of epulides under local anaesthesia. In our case, we decided for general anaesthesia and securing the airway by ETT in view of possibility of oral bleeding and aspiration.

Anaesthesia was maintained with oxygen + nitrous oxide and sevoflurane using Jackson Ree’s circuit, inj. vecuronium was used as muscle relaxant and fentanyl as analgesic. No significant bleeding was observed.

**Figure 3:**
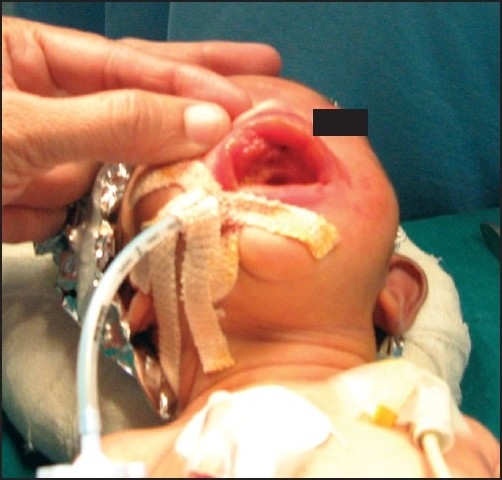
Post operative image of oral cavity

After excision of mass, there was no residual deformity and mouth opening of patient was absolutely normal [Figure 3]. Therefore, after reversal with inj. neostigmine and glycopyrrolate baby was extubated uneventfully.

In conclusion, epulis presents as a real challenge to anaesthesiologist for mask ventilation but intubation is possible as these masses have small pedicles and can be displaced during laryngoscopy. Assessment of an airway is mandatory for better airway control and safe endotracheal intubation. Prior to anaesthetising such babies we should also keep in mind remote possibility of surgical airway if required as a last life-saving measure as ventilation is difficult.
